# IL-35 subunit EBI3 alleviates bleomycin-induced pulmonary fibrosis via suppressing DNA enrichment of STAT3

**DOI:** 10.1186/s12931-021-01858-x

**Published:** 2021-10-28

**Authors:** Donghong Chen, Guofeng Zheng, Qing Yang, Le Luo, Jinglian Shen

**Affiliations:** 1 Department of Respiratory Medicine, The Fourth Affiliated Hospital of China Medical University/China Medical University, Seven South Road, Shenyang, 110005 Liaoning China; 2 Emergency Department, The Fourth Affiliated Hospital of China Medical University/China Medical University, Seven South Road, Shenyang, 110005 Liaoning China; 3 Shanghai Yunhao Biotech Center, Shanghai, 200000 China

**Keywords:** Pulmonary fibrosis, IL-35, STAT1/STAT4, STAT3, DNA enrichment

## Abstract

**Background:**

IL-35 subunit EBI3 is up-regulated in pulmonary fibrosis tissues. In this study, we investigated the pathological role of EBI3 in pulmonary fibrosis and dissected the underlying molecular mechanism.

**Methods:**

Bleomycin-induced pulmonary fibrosis mouse model was established, and samples were performed gene expression analyses through RNAseq, qRT-PCR and Western blot. Wild type and EBI3 knockout mice were exposed to bleomycin to investigate the pathological role of IL-35, via lung function and gene expression analyses. Primary lung epithelial cells were used to dissect the regulatory mechanism of EBI3 on STAT1/STAT4 and STAT3.

**Results:**

IL-35 was elevated in both human and mouse with pulmonary fibrosis. EBI3 knockdown aggravated the symptoms of pulmonary fibrosis in mice. EBI3 deficiency enhanced the expressions of fibrotic and extracellular matrix-associated genes. Mechanistically, IL-35 activated STAT1 and STAT4, which in turn suppressed DNA enrichment of STAT3 and inhibited the fibrosis process.

**Conclusion:**

IL-35 might be one of the potential therapeutic targets for bleomycin-induced pulmonary fibrosis.

## Background

Idiopathic pulmonary fibrosis is an age-related, chronic progressive interstitial lung disease of unknown cause and has few treatment options [1]. As a chronic disease worldwide, 10 to 60 out of 100,000 people are affected every year by idiopathic pulmonary fibrosis and with 34,000 new cases annually in the United States [2]. Idiopathic pulmonary fibrosis makes healthy lung tissue inelastic and scarring and result in many inflammatory reactions occur, therefore it has poor prognosis [3]. As the fibrous tissue replaces the normal lung tissues, the lungs cannot effectively transfer oxygen to the blood, and the patients will eventually suffer from death risks such as respiratory failure [4]. So far, there is few effective treatment for idiopathic pulmonary fibrosis except for lung transplantation [5]. Recently, the pneumonia caused by the COVID-19 has also caused researchers to pay attention to the risk of sequelae of pulmonary fibrosis in a set of severely ill patients [6].

Overexpression and deposition of collagen are important pathological features of idiopathic pulmonary fibrosis [7]. Bleomycin is an anti-tumor chemotherapeutic agent, which can cause subacute alveolitis and pulmonary fibrosis as the side effects [8]. Bishop et al. reported that the collagen synthesis rate of the animal’s pulmonary artery wall was significantly increased after 14 days of trachea instillation, collagen degradation was inhibited, and collagen in the blood vessel wall was deposited significantly high [9]. Cytokine interleukin 35 (IL-35) is a member of the IL-12 family, which is produced by regulatory T cells and plays important role in immunosuppression [10]. IL-35 is a dimeric protein composed of IL-27β chain and IL-12α chain and, encoded by two independent genes IL-12A and EBI3, respectively [11]. IL-35 inhibits T cell proliferation by inducing cell cycle arrest in the G1 phase [12]. Recent studies demonstrated that compared with the alveolar lavage fluid of non-tuberculosis patients, IL-35 levels in tuberculosis patients are significantly higher, IL-35 levels in Tubercle Bacillus (TB) positive patients were significantly higher than that in TB negative patients, in addition, L-35 was significantly lower in the cured patients than un-cured patients [13, 14].

Our previous results showed that the expression level of IL-35 subunit EBI3 was significantly increased in pulmonary fibrosis tissues induced by bleomycin treatment [15]. In this study, we used EBI3 knockout (KO) mice to prove that IL-35 inhibits the progression of pulmonary fibrosis, as well as cellular models, and dissected the underlying molecular mechanism. Our finding indicated that IL-35 might be one of the potential therapeutic targets for bleomycin-induced pulmonary fibrosis.

## Materials and methods

### Reagents

The bleomycin sulfate (B5507), human IL-6 (H7416) and IL-35 (SRP8053) recombinant proteins were ordered from Sigma-Aldrich (St. Louis, MO, USA).

### Human studies

Seventeen patients with pulmonary fibrosis and 18 healthy controls were recruited in this study. The study protocol of human has been approved by the ethics committee of the Fourth Affiliated Hospital of China Medical University. All participants gave written informed consent before taking part in the study. The clinical information of participates, including gender, age, forced expiratory volume in 1 s (FEV1): 105% predicted, and forced vital capacity (FVC): 103% pred were listed in Table 1. The expression levels of EBI3 and Il27a in the peripheral blood mononuclear cells of patients with pulmonary fibrosis and healthy controls were determined by qRT-PCR as described below.

**Table 1:** Patient information

Sample name	Disease	Gender	Age	FEVI%pred	FVC%pred	Drug for antifibrotics
PBMC#1	Pulmonary fibrosis	Female	47	55.8	84.2	No
PBMC#2	Pulmonary fibrosis	Male	45	77.2	83	Esbriet
PBMC#3	Pulmonary fibrosis	Male	51	63.9	80.6	No
PBMC#4	Pulmonary fibrosis	Female	30	81.9	75.6	Esbriet
PBMC#5	Pulmonary fibrosis	Male	42	76.3	80.9	No
PBMC#6	Pulmonary fibrosis	Female	33	81.3	71.2	No
PBMC#7	Pulmonary fibrosis	Female	38	77.8	85.7	No
PBMC#8	Pulmonary fibrosis	Female	54	57.1	76.5	No
PBMC#9	Pulmonary fibrosis	Male	55	67.6	73.6	No
PBMC#10	Pulmonary fibrosis	Male	38	88	72.2	Esbriet
PBMC#11	Pulmonary fibrosis	Male	20	69.5	71.8	No
PBMC#12	Pulmonary fibrosis	Female	49	62.2	75.4	No
PBMC#13	Pulmonary fibrosis	Female	29	62.2	70.7	No
PBMC#14	Pulmonary fibrosis	Male	58	79.9	85.6	No
PBMC#15	Pulmonary fibrosis	Female	42	74	81.6	No
PBMC#16	Pulmonary fibrosis	Male	48	76.7	80.6	No
PBMC#17	Pulmonary fibrosis	Male	35	60.1	80.2	No
PBMC#18	Healthy control	Female	51	95.4	96.1	No
PBMC#19	Healthy control	Male	29	94.6	95.4	No
PBMC#20	Healthy control	Female	34	96.4	96.9	No
PBMC#21	Healthy control	Female	51	96.1	96.6	No
PBMC#22	Healthy control	Male	36	88.9	90.2	No
PBMC#23	Healthy control	Male	41	97.2	97.7	No
PBMC#24	Healthy control	Male	51	95.4	96.1	No
PBMC#25	Healthy control	Male	55	98.3	98.7	No
PBMC#26	Healthy control	Male	32	87.6	89	No
PBMC#27	Healthy control	Female	42	89.6	90.9	No
PBMC#28	Healthy control	Female	36	92.9	93.8	No
PBMC#29	Healthy control	Female	32	89.6	90.9	No
PBMC#30	Healthy control	Female	48	87.3	88.7	No
PBMC#31	Healthy control	Male	58	93.2	94.1	No
PBMC#32	Healthy control	Male	55	94	94.8	No
PBMC#33	Healthy control	Female	53	86.1	87.7	No
PBMC#34	Healthy control	Female	52	85	86.7	No
PBMC#35	Healthy control	Male	38	90.2	91.5	No

### Animal studies

C57BL/6J wild type (WT) mice and EBI3 KO mice with B6 background were purchased from GemPharmatech (Nanjing, China). All animals were maintained under pathogen-free conditions and monitored daily. Mice were housed in standard mouse cages at 24 °C and fed with water and standard rodent chow diet. All experiments were performed following the experimental protocol for animal study approved by the Animal Care and Use Committee of The Fourth Affiliated Hospital of China Medical University. Pulmonary fibrosis model was established by intradermal injection with bleomycin to WT and EBI3 KO mice to induce lung fibrosis, according to the protocol described previously [16, 17]. In brief, mice were intradermally injected with 50 μl PBS or bleomycin (6 U/kg/day), 5 times per week, for 4 weeks, to create the pulmonary fibrosis model. At day 28, mice were euthanized, and lung samples were harvested to assess fibrosis using molecular and biochemical methods.

### Lung function analysis

After 4 weeks PBS/bleomycin treatment, the lung functions, including resistance, elastance and compliance, of WT and EBI3 KO mice were measured by using FlexiVent (SCIREQ Scientific Respiratory Equipment Inc., Montréal, Canada), following the manufacture’s instruments. The concentration of hydroxyproline in lung tissues of different treated groups was detected using the Hydroxyproline Assay Kit (ab222941, Abcam, Cambridge, UK).

### Cell culture and treatment

The mouse primary lung epithelial cells were obtained from American Type Culture Collection (ATCC, Manassas, USA), and culture in small airway growth medium (SAGM) supplemented with 1% fetal bovine serum as previously described [18]. Lung epithelial cells were co-stimulated with IL-35 (5 ng/ml) for 0, 15, 30, and 60 min, or co-treated with IL-6 (10 ng/ml), and then harvested for Western blot and CHIP qPCR analyses.

### RNAseq analysis

Lung tissues from PBS or bleomycin treated mice were collected for RNA extraction with TRIzol reagent and subjected to RNA-sequencing analysis (n = 3 for each group). RNAseq was performed by BGI Group (Shenzhen, China) and data were analyzed using the RSEM software package. In brief, Illumina HiSeq 2500 system was used to perform RNA sequencing by using the latest versions of sequencing reagents and flow cells, which provide up to 300 GB of sequence information per flow cell. The raw reads were mapped to the mm10 reference genome (build mm10) by using Bowite. RSEM software package was used to quantify the gene expression levels. The cut-off pf RNA integrity number (RIN) value was set as ≥ 7.0 for sample inclusion and downstream processing of RNA sequencing analysis.

### qRT-PCR and chromatin immunoprecipitation (CHIP) qPCR

Total RNA was extracted from 100 mg of tissue or 2 × 10^6^ primary lung epithelial cells using Trizol Reagent (Thermo Fisher Scientific, Waltham, USA). The complementary DNA (cDNA) was synthesized using the Thermo Fisher First-strand cDNA Synthesis Kit (Waltham, USA) and used as template and Applied Biosystems™ 7500 Real-Time PCR System was used to perform qPCR analysis. CHIP qPCR of Col1a2 and Col3a1 in mouse primary lung epithelial cells was performed as previously described [19]. Briefly, 3 × 10^7^ cells were used to perform CHIP. Formaldehyde was used to cross-link proteins to the genomic DNA for 20 min, which was terminated by adding glycine. Cells were suspended in CHIP lysis buffer and sonicated to shear DNA to an average size of 200–1000 bp. DNA concentration and fragment size were determined by Nanodrop and agarose gel electrophoresis. 5 μg STAT1 or STAT4 antibody was added to purified DNA and incubated at 4 °C for 2 h. Added 50 µl of blocked protein A/G beads to all samples and IP overnight with rotation at 4 °C. The DNA was immune-precipitated and purified by using phenol:chloroform extraction method. qRT-PCR was used to analyze the enrichment of Col1a2 and Col3a1 DNA. Predesigned Taq-man probe-based primers were purchased from Invitrogen (Carlsbad, USA). The sequences of qPCR primers were listed below:

Mouse EBI3 sense 5ʹ-CTCTCAAGTACCGACTCCGCTA-3ʹ;

Mouse EBI3 antisense 5ʹ-CTGAGCTGACACCTGGATGCAA-3ʹ;

Human EBI3 sense 5ʹ-CTGGATCCGTTACAAGCGTCAG-3ʹ;

Human EBI3 antisense 5ʹ-CACTTGGACGTAGTACCTGGCT-3ʹ;

Mouse Col1a2 sense 5ʹ-TTCTGTGGGTCCTGCTGGGAAA-3ʹ;

Mouse Col1a2 antisense 5ʹ-TTGTCACCTCGGATGCCTTGAG-3ʹ;

Mouse Col3a1 sense 5ʹ-GACCAAAAGGTGATGCTGGACAG-3ʹ;

Mouse Col3a1 antisense 5ʹ-CAAGACCTCGTGCTCCAGTTAG-3ʹ;

Mouse Lox sense 5ʹ-CATCGGACTTCTTACCAAGCCG-3ʹ.

Mouse Lox antisense 5ʹ-GGCATCAAGCAGGTCATAGTGG-3ʹ;

Mouse FN1 sense 5ʹ-CCCTATCTCTGATACCGTTGTCC-3ʹ;

Mouse FN1 antisense 5ʹ-TGCCGCAACTACTGTGATTCGG-3ʹ;

Mouse TIMP1 sense 5ʹ-TCTTGGTTCCCTGGCGTACTCT-3ʹ;

Mouse TIMP1 antisense 5ʹ-GTGAGTGTCACTCTCCAGTTTGC-3ʹ;

Mouse MMP13 sense 5ʹ-GATGACCTGTCTGAGGAAGACC-3ʹ;

Mouse MMP13 antisense 5ʹ-GCATTTCTCGGAGCCTGTCAAC-3ʹ;

### Western blot

The IL-35 or IL-35 + IL-6 treated primary lung epithelial cells were lysed using the RIPA Lysis Buffer ordered from Beyotime (Shanghai, China) and added protease inhibitor cocktails freshly (Sigma-aldrich, St. Louis, USA). Total protein concentration was determined by Pierce™ BCA Protein Assay Kit (Thermo Fisher Scientific, Waltham, USA). The expression level of target proteins was determined by Western blot as previously described [20]. Antibodies used in this study were listed below: rabbit monoclonal antibody for phospho-Stat1 (Tyr701) (D4A7, 1:1000 dilution), Stat1 (D1K9Y, 1:1000 dilution), phospho-Stat3 (Tyr705) (D3A7, 1:1000 dilution), Stat3 (D3Z2G, 1:1000 dilution) and Stat4 (C46B10, 1:1000 dilution) were purchased from Cell Signaling Technology (Danvers, USA). Antibody for p-Stat4 (E-2) (sc-28296, 1:1000 dilution) was ordered from Santa Cruz Biotechnology, Inc. (Dallas, USA).

### Immunohistochemistry staining

The lung tissues from WT or EBI3 KO mice were formalin fixed and paraffin-embedded, sectioned at 5 to 6 μm, deparaffinized, and rehydrated. Masson Trichrome staining was performed following the standard laboratory procedures. Optical light microscopy (Leica, Wetzlar, Germany) was used to take the images of sections, and Masson’s Trichrome staining was carried out by using the Trichrome Stain Kit (Abcam, Cambridge, UK), following the protocol provided by the manufacturer. Ashcroft score was determined according the protocol published by Hübne et al. [21].

### ELISA

The serum of mice was collected at day 0, 14, and 28 after the bleomycin treatment. The concentration of EBI3 in serum was determined by using the IL-27 Mouse ELISA Kit (Invitrogen, Carlsbad, USA). Serum from 18 health donors and 18 patients with pulmonary fibrosis was used to detect the concentration of EBI3 by using Human IL-27 ELISA Kit (Invitrogen, Carlsbad, USA), following the protocols provided by manufacture. All participants gave written informed consent before taking part in the study.

### Statistical analysis

GraphPad Prism8.0 was used for all statistical analyses in this study. The unpaired 2-tailed *t* test or one-way ANOVA analysis was used for the comparison of parameters between two groups or multiple groups, respectively. The data were represented mean ± standard deviation (SD). The level of significance was set at *P* < 0.05.

## Results

### EBI3 is up-regulated in bleomycin-induced pulmonary fibrosis mouse model

**Fig. 1:**
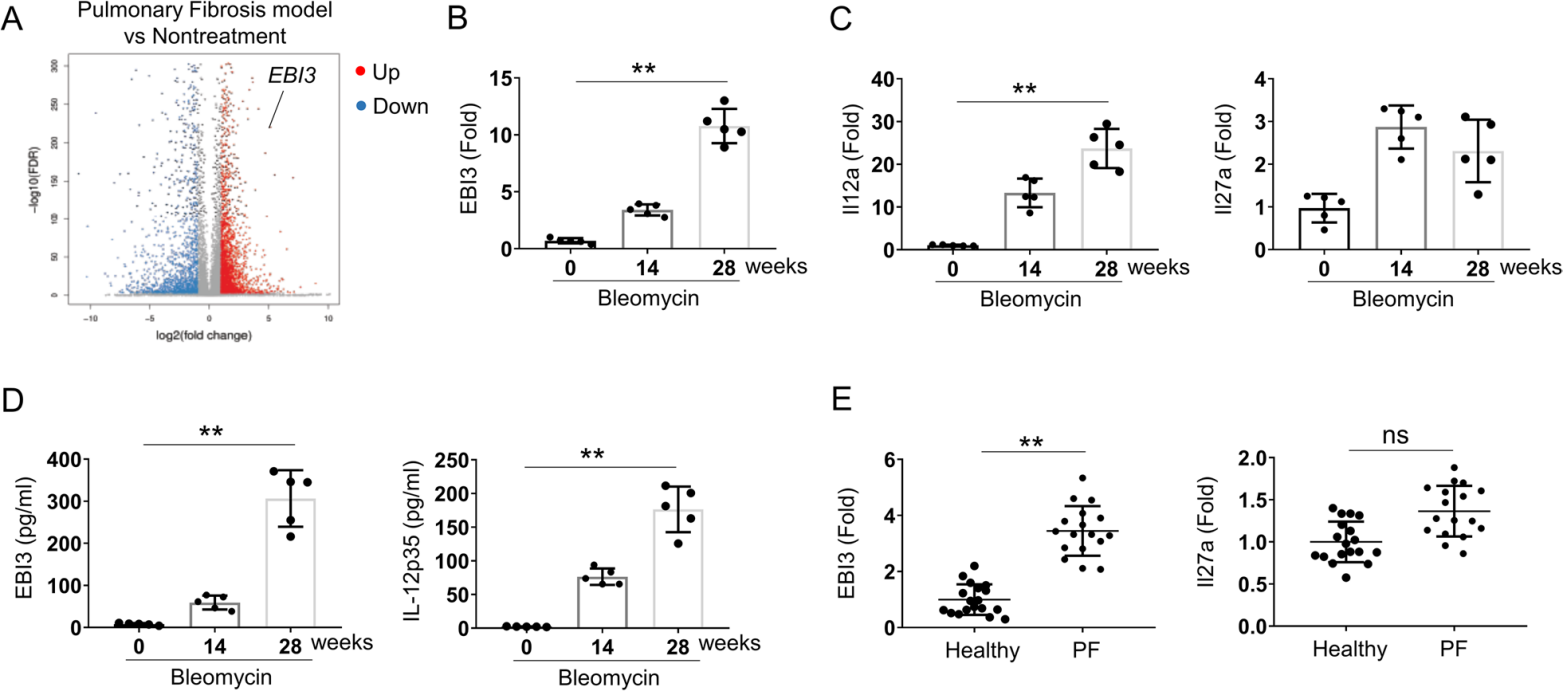
The expression of EBI3 was upregulated in bleomycin-induced pulmonary fibrosis model. **A** Volcano graph showing the differentially expressing genes in the lung between nontreated and bleomycin-induced pulmonary fibrosis model mice. **B**, **C** qPCR analysis of EBI3 (**B**), Il12a and Il27a (**C**) in the lung tissues from nontreated and bleomycin-induced pulmonary fibrosis model mice. Actin was used as loading control and for the relative normalization (n = 5). **D** The serum was collected at the indicated time points. The protein level of EBI3 and IL-12p35 were measured by ELISA (n = 5). **E** qPCR analysis of EBI3 and Il27a in the peripheral blood mononuclear cells from the healthy donors (n = 18) or patients with pulmonary fibrosis (n = 17). Data are representative of three independent experiments. Data are presented as mean ± SEM. Significance was determined by two-tailed Student’s t test. **P < 0.01

The C57BL/6J WT mice were intradermally injected with bleomycin to induce pulmonary fibrosis as previously described [22]. To profile the transcriptional features of pulmonary fibrosis, the lung tissues of PBS and bleomycin treated mice were analyzed by RNAseq (Fig. 1A). A lot of genes were increased in pulmonary fibrosis tissues, and EBI3, a subunit of the cytokine IL-35, was one of highest up-regulated genes (Fig. 1A). The up-regulation of EBI3 was confirmed by qRT-PCR, and the expression level of EBI3 was increased gradually with the prolongation of bleomycin treatment (day 0, 14, and 28) (Fig. 1B). We next detected the expressions of IL-12p35 and IL-27p28 with qPCR. We found another IL-35 subunit IL-12p35 was also upregulated, indicating Blemycin induced Il-35 production (Fig. 1C). However, Il27a was only weakly upregulated, implying IL-27 might only involve in this processing (Fig. 1C). Moreover, upon bleomycin treatment, the serum EBI3 and IL-12p35 protein level was increased too (Fig. 1D). IL-27 has been shown to be produced by human monocytic cells primed with IFN-γ and in response to a second stimulus such as LPS. IL-35 displayed a regulatory function in immune response, which potentially inhibited IL-27p28 expression. We further investigated the expression level of EBI3 in the peripheral blood mononuclear cells from the healthy donors or patients with pulmonary fibrosis, and the qRT-PCR result showed EBI3 mRNA level was significantly increased in patients with pulmonary fibrosis compared to that in healthy donors (Fig. 1E and Table 1). However, the patients with pulmonary fibrosis did not display an increase in mRNA level of Il27a induction. All these data suggested that cytokine IL-35 was increased in lung tissues with pulmonary fibrosis of both human patients and mouse model, indicating IL-35 might involve into the progression of pulmonary fibrosis.

### EBI3 deficiency aggravates the symptoms of bleomycin-induced pulmonary fibrosis

**Fig. 2:**
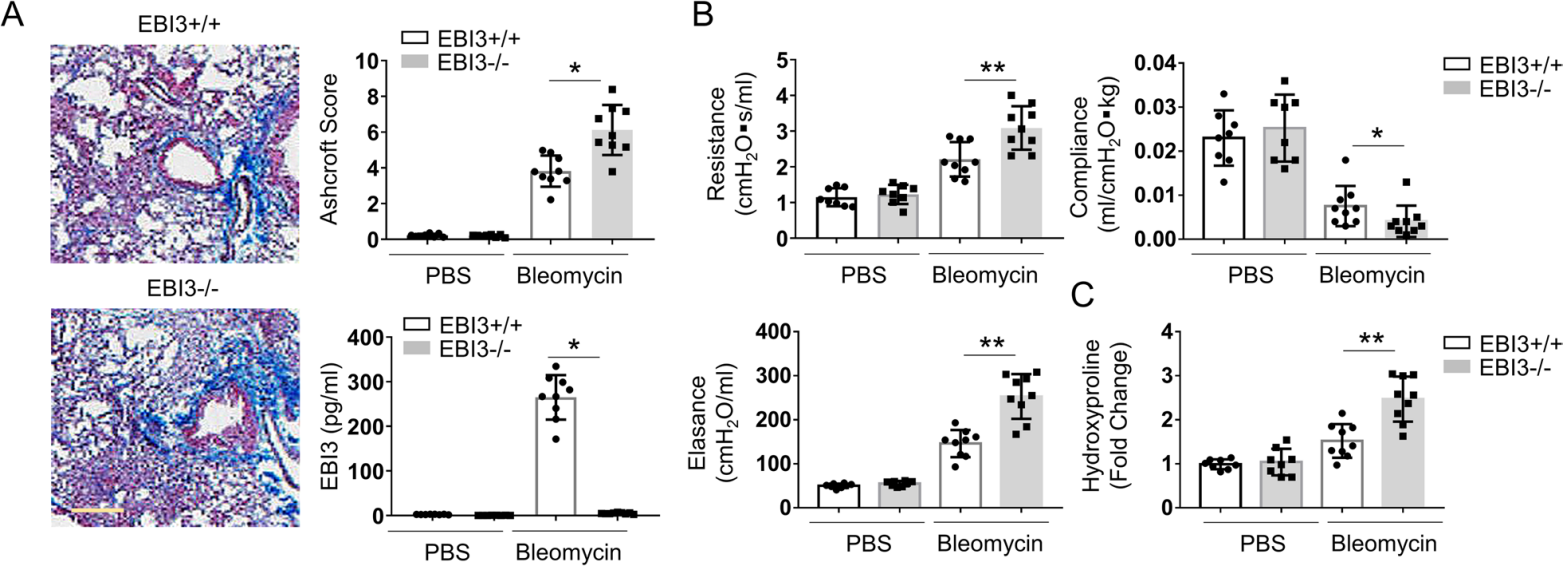
EBI3 deficiency aggravated the symptoms of bleomycin-induced pulmonary fibrosis. **A** Lung sections of bleomycin treated WT and EBI3 KO mice were stained with Masson Trichrome. Scale bar 200 μm. The serum was collected from these two mice. The protein level of EBI3 was measured by ELISA (n = 9). **B** Lung function measurements including resistance, elastance and compliance were measured in all four groups using FlexiVent (n = 9). **C** The lung hydroxyproline was evaluated in bleomycin treated WT and EBI3 KO mice (n = 9). Data are representative of three independent experiments. Data are presented as mean ± SEM. Significance was determined by two-tailed Student’s t test. *P < 0.05 and **P < 0.01

To explore the pathological role of IL-35 in pulmonary fibrosis, WT and EBI3 KO (EBI3−/−) mice were used to assess lung function after 4 weeks bleomycin exposure. The ELISA assay revealed a deficiency of EBI3 in EBI3 KO mice (Fig. 2A). Masson Trichrome staining data showed that there was more pulmonary fibrosis accumulation in EBI3 KO mice relative to that in WT mice, and Ashcroft score of KO mice was significantly higher than WT control (Fig. 2A). Bleomycin exposure significantly increased the lung tissue resistance and elastance in both WT and EBI3 KO mice, interestingly, with bleomycin treatment the resistance and elastance in EBI3 KO mice were significantly higher than WT mice (Fig. 2B). In contrast, lung compliance was decreased in both WT and KO mice after bleomycin exposure, and it was further decreased in EBI3 KO mice (Fig. 2B). Hydroxyproline is an important biomarker for pulmonary fibrosis, which increased in the lung tissues of bleomycin exposed mice, and the highest hydroxyproline level was observed in EBI3 KO mice (Fig. 2C). The above lung function data indicated that EBI3 deficiency aggravated the symptoms of bleomycin-induced pulmonary fibrosis in mouse model.

### EBI3 deficiency enhances the expressions of extracellular matrix (ECM)-associated genes in bleomycin-induced pulmonary fibrosis

**Fig. 3:**
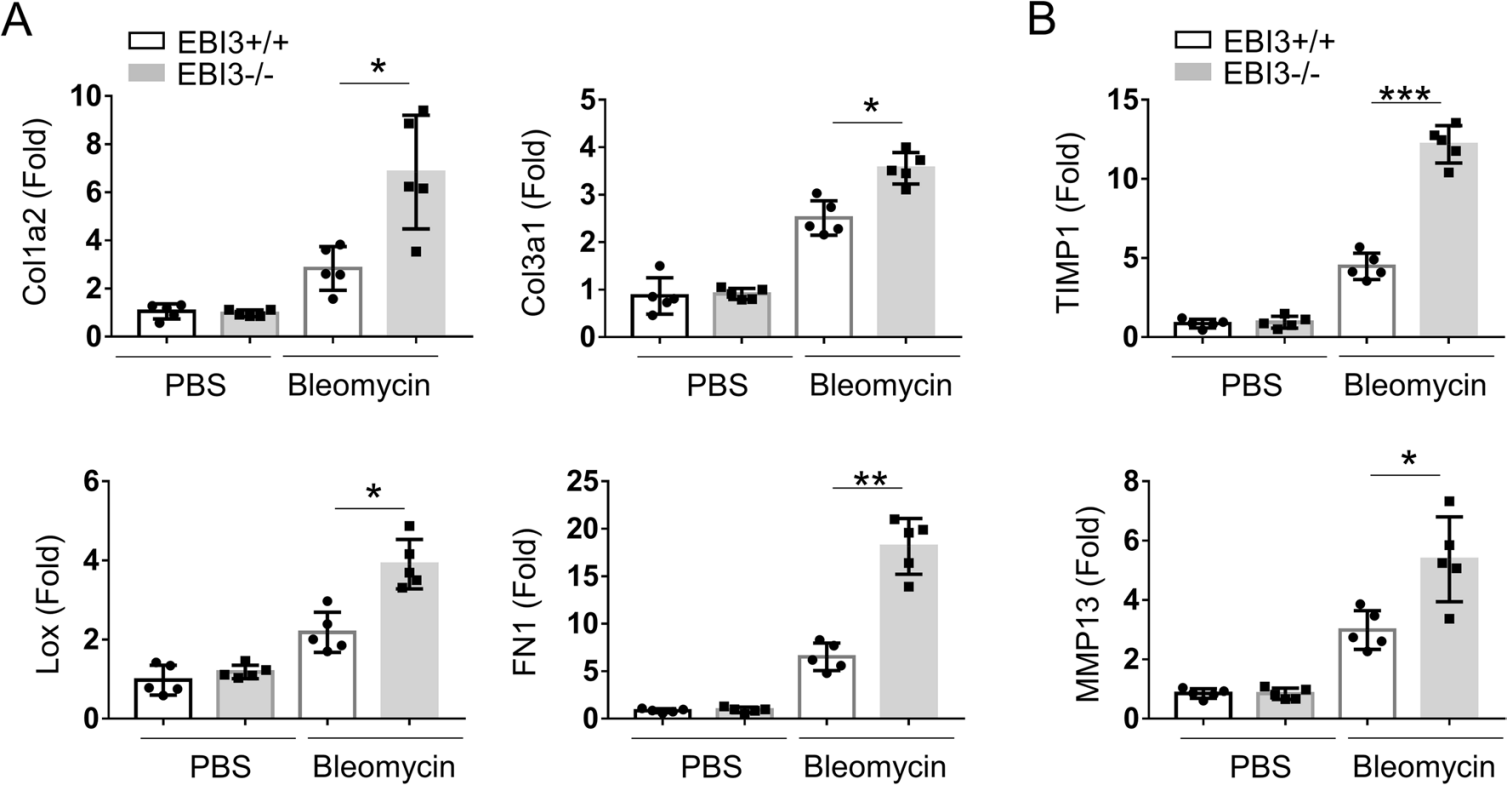
EBI3 deficiency enhanced the expressions of ECM-associated genes in bleomycin-induced pulmonary fibrosis. **A** qRT-PCR assay of ECM gene transcripts including Col1a2, Col3a1, lox and Fn1 (n = 5). **B** mRNA levels of Mmp13, Timp1 which was involved in ECM remodeling and production were measured by qPCR (n = 5). Data are representative of three independent experiments. Data are presented as mean ± SEM. Significance was determined by two-tailed Student’s t test. *P < 0.05, **P < 0.01 and ***P < 0.005

In order to further evaluate the pulmonary fibrosis induced by bleomycin, we performed qRT-PCR to analyze the expression of fibrosis-related genes in the lung tissues of WT and EBI3 KO mice. In comparison with PBS group, the ECM-associated genes, including Col1a2, Col3a1, Lox, and Fn1, were significantly increased in both WT and EBI3 KO mice after bleomycin exposure (Fig. 3A). Moreover, the Genes ECM production and reprogramming-related genes, TIMP1 and MMP13, were increased significantly too (Fig. 3B).

### IL-35 inhibits ECM-associated genes via competitively identifying STAT3 binding sites

**Fig. 4:**
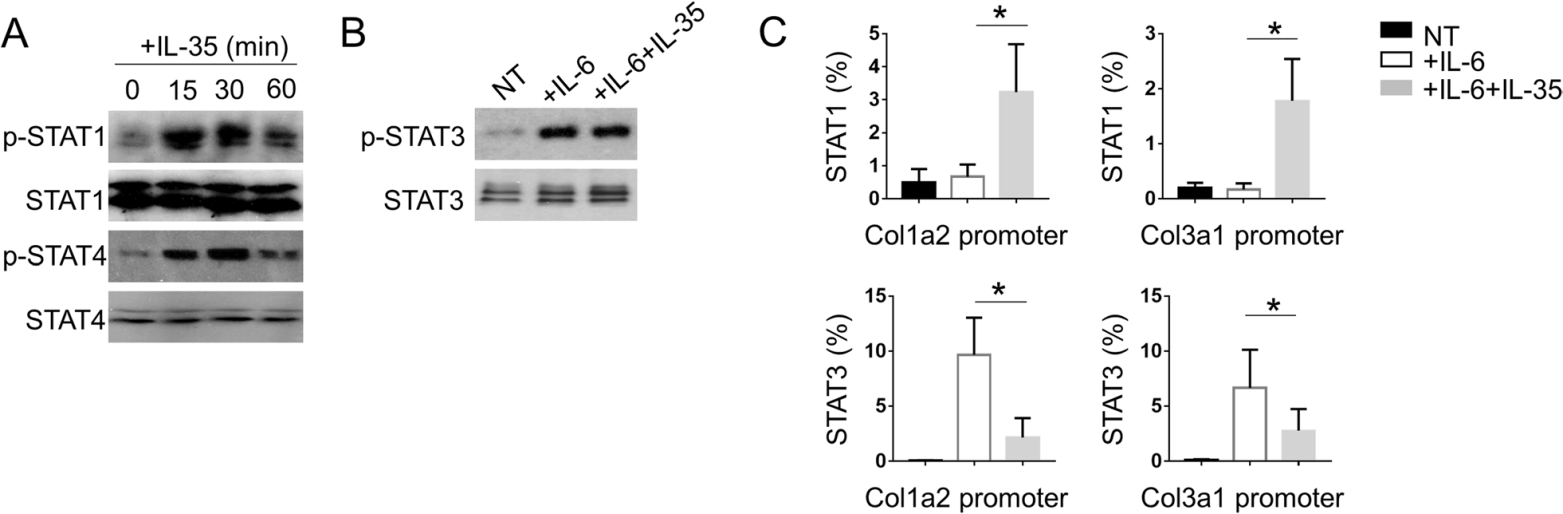
IL-35 inhibited ECM-associated genes via competitively identifying STAT3 binding sites. **A** Lung epithelial cells were stimulated with IL-35 (5 ng/ml) for indicated time points, and phosphorylation of STAT1 and STAT4 were measured by WB. **B** Lung epithelial cells were co-stimulated with IL-35 (5 ng/ml) and IL-6 (10 ng/ml), and phosphorylation of STAT3 were measured by WB. **C** CHIP qPCR assay for evaluating the enrichment of STAT1 and STAT4 on the promoters of Col1a2 and Col3a1. Input was used as the relative normalization (n = 3). Data are representative of three independent experiments. Data are presented as mean ± SEM. Significance was determined by two-tailed Student’s t test. *P < 0.05

IL-35 has been reported to induce the phosphorylation of STAT1 and STAT4 in T cells [23], to investigate whether the STATs was also activated by IL-35 in lung tissues, we treated the primary lung epithelium with IL-35 to detect the activation level of these two molecules through Western blot. The results showed that IL-35 induced the phosphorylation of both STAT1 and STAT4 in primary lung epithelium (Fig. 4A). Next, we performed CHIP qPCR assay to evaluate the enrichment of STAT1 and STAT4 on the promoters of Col1a2 and Col3a1. Although IL-35 did not affect the activation level of STAT3 induced by IL-6 (Fig. 4B), IL-35-induced the activation of STAT1 could competitively bind to the binding site of STAT3 and then inhibit the expression of Col1a2 and Col3a1 (Fig. 4C). These results suggested that IL-35 inhibited ECM-associated genes through competitively identifying STAT3 binding sites in primary lung epithelium.

## Discussion

Idiopathic pulmonary fibrosis is a chronic progressive interstitial lung disease that caused by unknown factors, so far, there has few treatment options for this disease [1]. In this study, we established the bleomycin-induced pulmonary fibrosis mouse model and performed transcriptional profile analysis using RNAseq. In comparison with control groups, IL-35 (EBI3) was one of the most up-regulated genes in the lung tissues of bleomycin-induced pulmonary fibrosis mice and patients with pulmonary fibrosis. Moreover, knockdown of IL-35 subunit EBI3 increased the expression of a variety of fibrosis-related genes and aggravated the symptoms of bleomycin-induced pulmonary fibrosis in mouse model. Mechanistically, IL-35 promotes the phosphorylation level of STAT1 and STAT4, and activated STAT1 and STAT4 competitively bind to the recognition region of STAT3 and inhibit the fibrosis process caused by STAT3. Our finding indicated that IL-35 might be one of the potential therapeutic targets for the treatment of pulmonary fibrosis.

Inflammatory responses play important role in the progression of pulmonary fibrosis. Cytokines exhibit significant pro-fibrotic activity, which have received the widespread attention [24–26]. As an archetypal type-2 cytokine, interleukin 4 (IL-4) has been reported function as a pro-fibrotic cytokine and significantly elevated in idiopathic pulmonary fibrosis [27]. Sempowski et al. demonstrated that IL-4 was the up-stream effector of TGF-β that play important role at inducing collagen synthesis from the fibroblasts [28]. Interleukin 13 (IL-13) is another fibrogenic cytokine that shares many properties with IL-4 in many fibrotic conditions [26, 29]. The current study strengthens their role of immunomodulation in the pathogenesis of fibrosis. Both IL-4 and IL-3 are related cytokines that regulate many aspects of inflammation and have been reported to involve into the inflammatory responses of pulmonary fibrosis [30, 31]. Interestingly, blocking or knockdown IL-13 could reduce the bleomycin- or Fluorescein isothiocyanate (FITC)-induced collagen deposition [32, 33]. IL-35 is a new member of IL-12 cytokine family, which has anti-inflammation effect and plays important role in immune suppression [34, 35]. In current study, we found that IL-35 expression level was significantly elevated in the lung tissues of patients with pulmonary fibrosis and bleomycin-induced pulmonary fibrosis mouse model. Importantly, knockdown of IL-35 resulted in lung function loss significantly, including increased lung resistance and elastance, and decreased lung compliance. The above pathological changes could be interpreted by the elevated fibrosis, including more Masson Trichrome staining and higher hydroxyproline concentration, and up-regulation of genes associated with fibrosis and ECM remodeling/production. Our finding suggested that IL-35 might be work as an anti-inflammation cytokine with the ability of reducing pulmonary fibrosis in mouse model.

STAT3, a widely expressed transcription factor, is activated during inflammatory reaction and contributes to the tissue injury and repair due to its positive roles in cell survival and proliferation [36, 37]. However, the persistent activation of STAT3 induces the overexpression of fibrotic and ECM-associated genes, and finally lead to fibrosis [38, 39]. In line with the previous findings, we found that STAT3 also could be activated by IL-6 in primary lung epithelial cells [40], co-treatment IL-6 with IL-35 did not inhibit the activation of STAT3 (Fig. 4B). Interestingly, IL-35 promotes the phosphorylation level of STAT1 and STAT4, which in turn competitively bind to the recognition region of STAT3, and then inhibit the fibrosis process caused by STAT3. However, there are limitations of this finding, because the interaction between IL-35/IL-6 and STAT3 was detected in lung epithelial cells instead of animal model, which need to be test in vivo in the following experiments. Nevertheless, bleomycin-induced mouse model is not biologically equivalent to human idiopathic pulmonary fibrosis. The IL-35 might be one of the potential therapeutic targets for pulmonary fibrosis induced by bleomycin, while only by obtaining strict and accurate clinical trial data we can conclude that this finding is also effective in clinical medicine. In the future studies, we will investigate the anti-pulmonary fibrosis effect of IIL-35 in vivo, and it is also interested to address the anti-fibrotic property of IL-35 is restricted to the lung or it is widely present in multiple organs, such as liver, kidney, and skin.

## Conclusion

IL-35 is up-regulated genes in the lung tissues pulmonary fibrosis, which plays an anti-pulmonary fibrosis role in cell and mouse models. Therefore, IL-35 might be one of the potential therapeutic targets for the treatment of pulmonary fibrosis.

## Data Availability

Data could be obtained upon request to the corresponding author.

## Abbreviations

IL-35: Interleukin 35
RIN: RNA integrity number
